# Improved Flexible Mechanical Delivery and Detachment System for Embolization Coils: Design, Modeling, and Experimental Evaluation

**DOI:** 10.7759/cureus.92059

**Published:** 2025-09-11

**Authors:** Oleh Nechyporuk, Mykola Nechyporuk

**Affiliations:** 1 Neurological Surgery, Brovary Multidisciplinary Clinical Hospital, Brovary, UKR

**Keywords:** aneurysm, endovascular thrombectomy, mechanical detachment mechanism, microcatheter navigation, microcoil delivery system

## Abstract

Dense and safe packing of intracranial aneurysms with embolization microcoils is critically important to prevent recurrence. While the properties of the coils themselves are widely studied, the mechanical characteristics of the delivery and detachment systems also significantly affect implantation quality. This study presents a novel system combining a dual-core stainless steel microcable and an outer delivery spiral with a flexible looped hinge detachment. The design improves flexibility and catheter control in tortuous anatomy. Bench testing demonstrated reduced bending stiffness, greater angular mobility, and immediate clean detachment without residual components. The prototypes withstood loads far exceeding clinically acceptable limits. The proposed system may improve endovascular embolization outcomes and serve as a foundation for further development.

## Introduction

Endovascular embolization with microcoils is a widely accepted method for the treatment of intracranial aneurysms. The success and safety of this procedure depend not only on the properties of the coils themselves [1,2], but also on the mechanical characteristics of the delivery and detachment systems [3]. Although commercial systems are generally effective, they may be limited in navigating tortuous vessels, sharp angiographic angles, complex aneurysm locations, or other anatomical difficulties [4]. Mechanical mismatch between the delivery system and the vascular anatomy may lead to reduced navigation stability, excessive load on the microcatheter, inaccurate coil placement, or even intraprocedural complications such as microcatheter kickback or aneurysm wall damage [5,6]. To address these challenges, new design solutions are required. One of the key objectives is to minimize the negative impact on the microcatheter during embolization while simultaneously improving operator control. At the same time, increasing the flexibility of delivery systems may negatively affect mechanical stability, detachment reliability, and overall safety. In this study, we present a new flexible mechanical delivery and detachment system for microcoil embolization, specifically designed for use in complex vascular anatomy. The system consists of a microcable placed inside a low-profile spiral and an improved microcoil detachment mechanism based on a looped hinge stabilized with a polyethylene thread. We describe the basic design principles, simulation results, and comparative mechanical characteristics relative to standard commercial devices. In addition, we provide calculations and preliminary experimental results that support the potential of the system for further clinical implementation.

## Technical report

Materials and methods

A central feature of the developed microcoil delivery system is the reduction of both axial and bending stiffness. This design enhances the flexibility of the distal segment, minimizes the size of rigid components in the microcoil fixation and detachment area (the so-called "lock"), and improves coil mobility at the connection point with the delivery system.

However, the increased maneuverability initially resulted in reduced axial stiffness and limited controllability during advancement through the microcatheter and while positioning the microcoil. Although high mobility facilitated coil deployment within the aneurysm, it complicated repositioning, and the initial fixation mechanism proved unreliable. These limitations were addressed through subsequent design modifications, and the current version is considered finalized. This study focuses on analyzing the strengths and limitations of this configuration and presents results from comparative mechanical modeling and preliminary experimental testing.

The interaction with the microcatheter was evaluated based on bending stiffness (EI) and compliance (C). To optimize these parameters, the intracranial segment of the system incorporates a dual-core stainless steel microcable (wire diameter 0.04 mm) with a distal loop [7,8]. 

**Figure 1:**
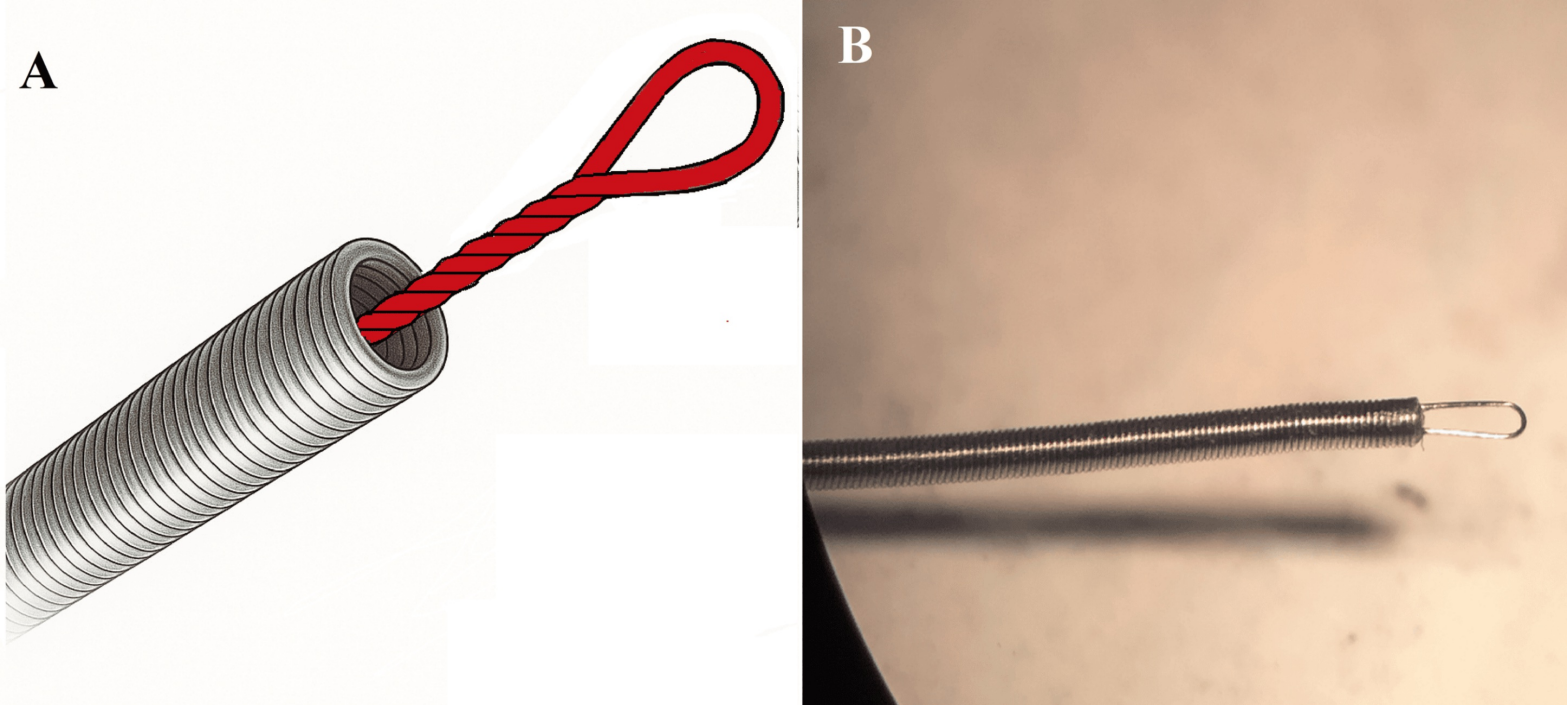
Images of the microcable (A) Technical sketch of the delivery spiral and microcable; and (B) Microscopic image of the delivery spiral with a microcable loop (taken with Carl Zeiss microscope (Ukraine). Image credit: Original photosketches edited by the authors in vector editor + paint (Inkscape, Inkscape Project, open-source software).

To ensure sufficient axial stiffness, the microcable is embedded within a 40 cm long delivery coil with an outer diameter of 0.32 mm, made of 0.04 mm nichrome wire. (Figures 1A, 1B)

**Figure 2:**
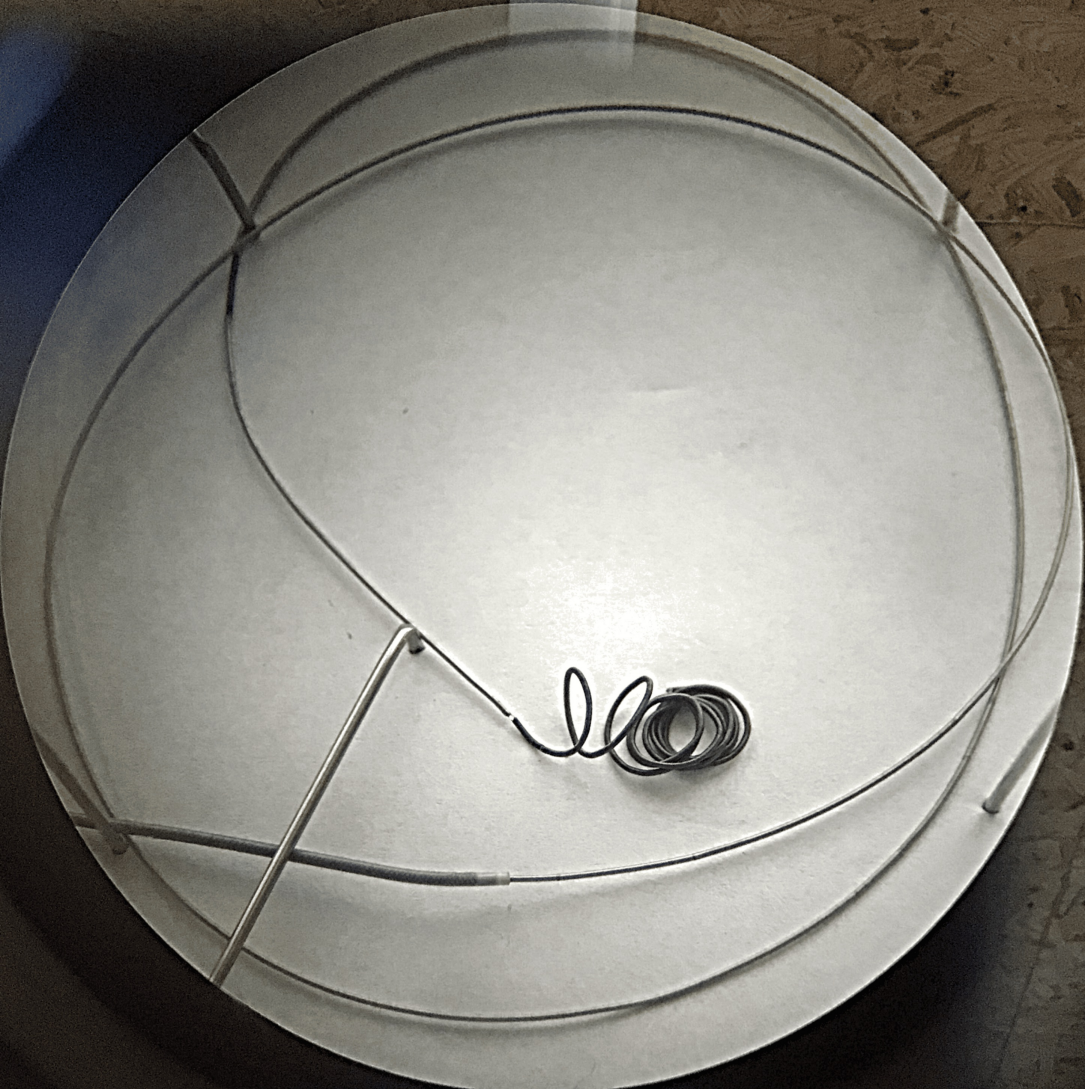
Microscopic image of the assembled microcoil, delivery system spiral, radiopaque marker, and the system located inside the microcatheter Image credit: Microscopic photo taken with the Carl Zeiss microscope (Ukraine).

A radiopaque marker was integrated via microwelding. While similar constructions have been used in other systems, the use of this specific configuration in a mechanical microcoil detachment system is likely novel (Figure 2)

Microcoil fixation and detachment mechanism

**Figure 3:**
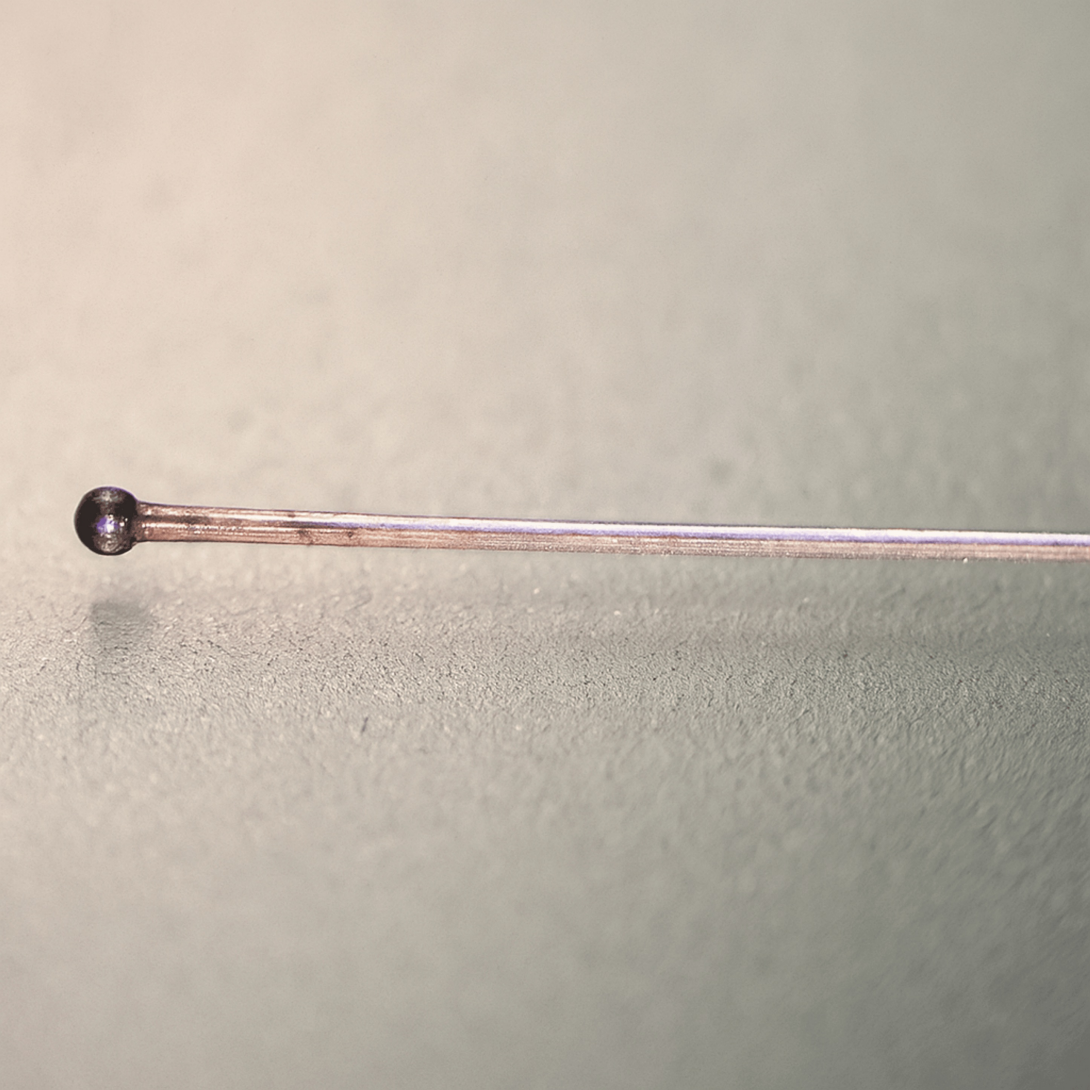
Microscopic image of a 0.08 mm diameter wire with a ball tip Image credit: Microscopic photo taken by the authors with the Carl Zeiss microscope (Ukraine).

The microcoil delivery system we developed features an original, structurally simplified fixation and detachment mechanism. In most modern commercial mechanical detachment systems, a wire approximately 0.08 mm in diameter with a ball tip at the distal end is used, which locks into the proximal end of the microcoil [3]. For technical comparison, we manufactured a similar ball-lock model but also proposed an alternative structural concept (Figure 3).

**Figure 4:**
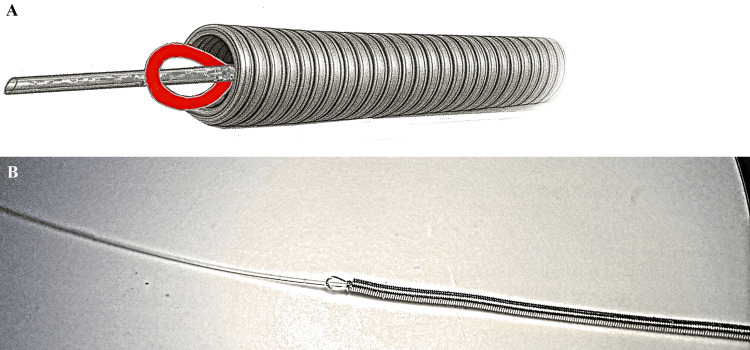
Technical sketches of the delivery system A) Technical sketch of the delivery system with a microcable loop and a plastic filament; B) Microscopic image of the delivery system with a microcable loop and a plastic filament. Image credit: Original photo sketches, edited by the authors in vector editor + paint (Inkscape, Inkscape Project, open-source software), and microscopic photo taken by the authors with the Carl Zeiss microscope (Ukraine).

In our system, fixation is achieved using a loop at the end of the delivery microcable. The microcoil is secured by a high-molecular weight polyethylene filament (fishing line) with a diameter of 0.06-0.08 mm, which passes through the aforementioned loop. This design significantly reduces the number of rigid components in the system while maintaining sufficient fixation reliability (Figures 4A, 4B)

System assembly: Connecting the microcoil to the delivery system

**Figure 5:**
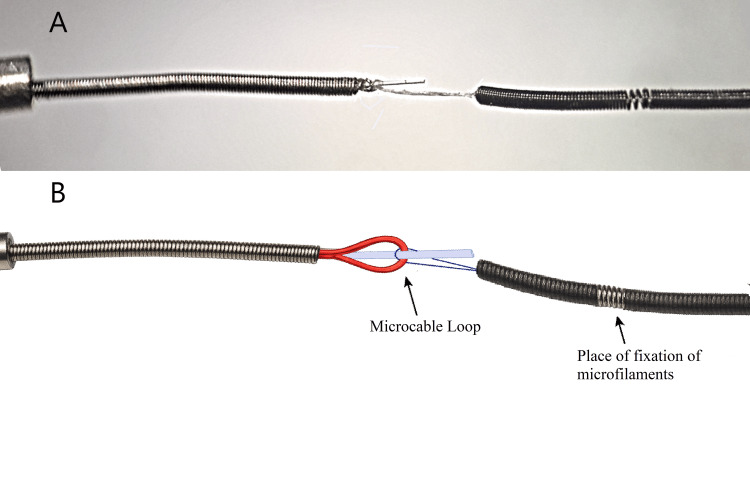
Images of the microcoil-to-delivery system A) Microscopic image of the microcoil-to-delivery system junction. The delivery system includes a delivery spiral, a plastic filament, and a microcable loop. The microcoil is shown with a fixed loop made of microfilaments and a designated area for photopolymer connection; B) Technical sketch of the microcoil-to-delivery system junction. The delivery system includes a delivery spiral, a plastic filament, and a microcable loop. The microcoil is shown with a fixed loop made of microfilaments and a designated area for photopolymer connection. Image credit: Microscopic photo taken by the authors with the Carl Zeiss microscope (Ukraine); Photo sketch edited from the original by the authors using vector editor + paint (Inkscape, Inkscape Project, open-source software).

System assembly was performed at a specialized workstation equipped with fixation, rotation, and cooling functions. The process involved connecting two structural loops: the loop of the delivery microcable, through which the polyethylene filament (0.06-0.08 mm) passed, and the loop formed from microfilaments at the proximal end of the microcoil. After securing the delivery system in the holder, the microfilament loop of the microcoil was passed through the wire loop and slipped over the polyethylene filament of the delivery system. In this configuration, all three components were joined into a single structure. Next, the microfilament loop was tensioned, and the polyethylene filament was inserted into the inner lumen of the microcoil (Figures 5A, 5B)

**Figure 6:**
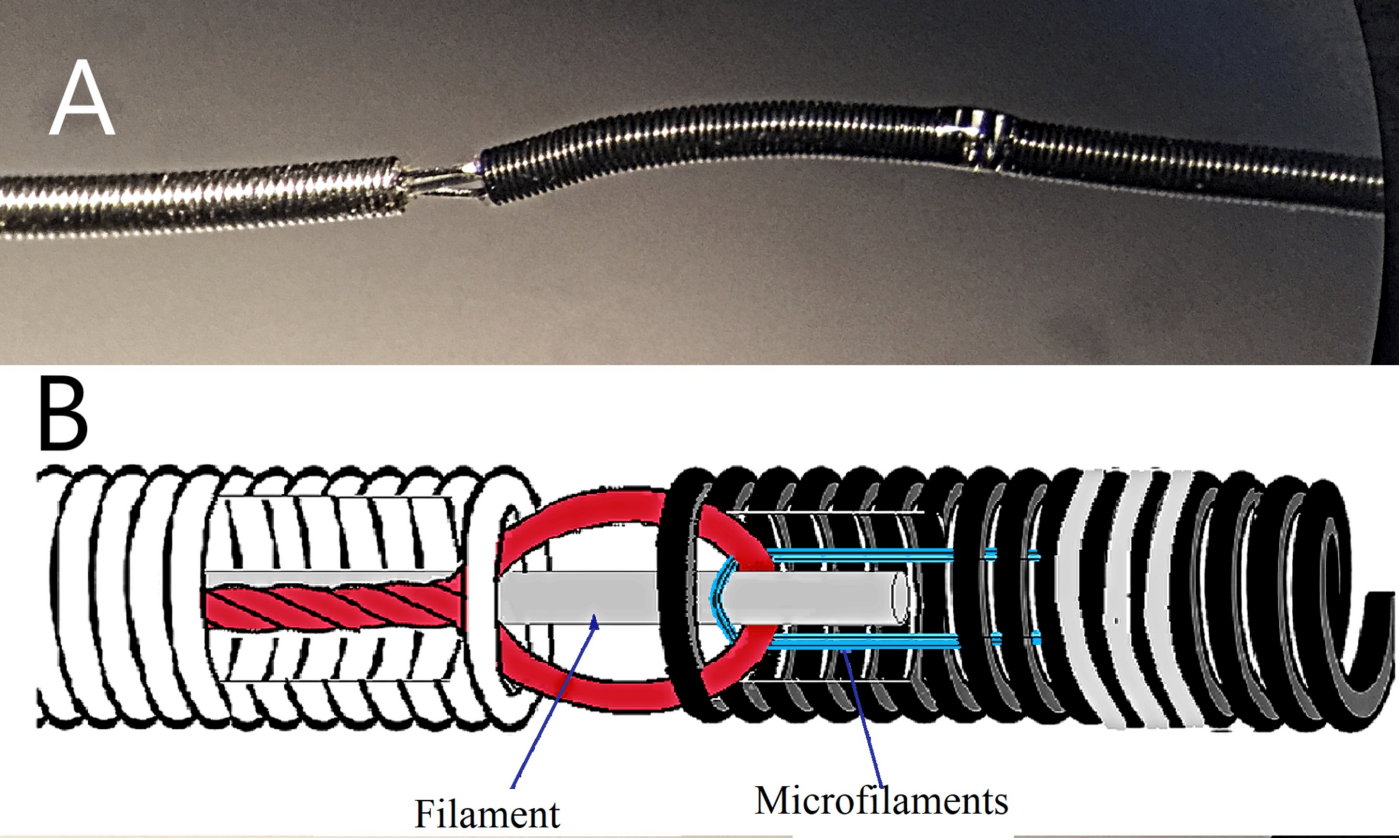
Images of the microcoil connected to the delivery system A) Microscopic image; B) Technical sketch Image credit: Microscopic photo taken by the authors with the Carl Zeiss microscope (Ukraine); and photo sketch edited by the authors from the original using vector editor + paint (Inkscape, Inkscape Project, open-source software).

The microcoil was then advanced toward the loop of the delivery microcable until mechanical resistance was encountered at a predefined axial force. The tensioned microfilaments were then fixed to the microcoil windings distal to the fixation zone using photopolymer acrylic. This mechanism functioned as a loop-shaped hinge, where the polyethylene filament served a dual role, providing both fixation and axial flexibility. The design ensured controlled mobility of the microcoil at the attachment point without the need for additional rigid components (Figures 6A, 6B)

This mechanism functioned as a loop-shaped hinge, where the polyethylene filament served a dual role - providing both fixation and axial flexibility. This design ensured controlled mobility of the microcoil at the attachment point without the need for additional rigid components.

Stability improvements of the system

Despite high maneuverability, the initial prototypes of the delivery system had insufficient axial stiffness and provided only two-point support for the microcoil on the microcable loop. The combination of a flexible filament and a hinge connection resulted in excessive coil mobility (hyperflexibility), which complicated its repositioning during implantation.

**Figure 7:**
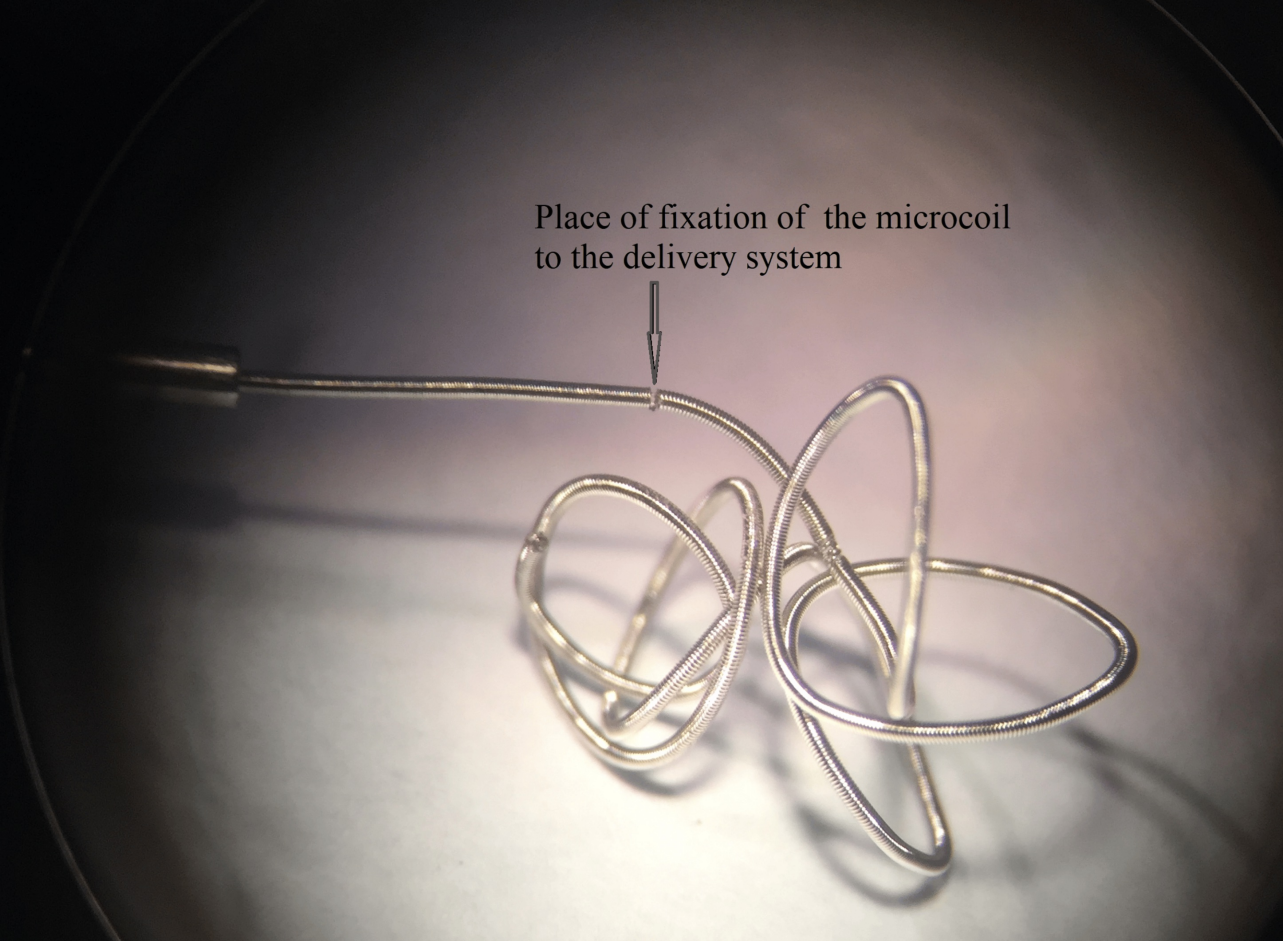
Microscopic image of a 3D-shaped platinum microcoil mounted on the delivery system Image credit: Microscopic photo taken by the authors with the Carl Zeiss microscope (Ukraine)

To address these issues, the loop was repositioned deeper into the delivery spiral. In the modified configuration, the microcoil rested simultaneously on the loop and on the turns of the delivery spiral, according to the “coil pressing against coil” principle. This modification improved overall stability without compromising the flexibility of the system (Figure 7).

**Figure 8:**
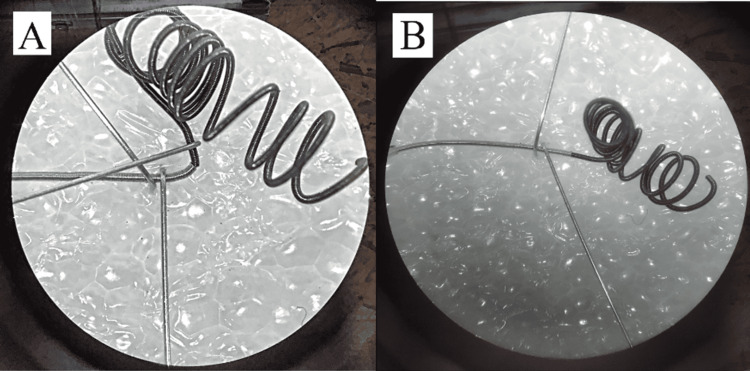
A) Microscopic image showing flexibility at the “lock” zone of the microcoil (deflection over 90°), and B) Microscopic image of the microcoil returning to its original shape after deflection in the “lock” zone Image credit: Microscopic photo taken by the authors with the Carl Zeiss microscope (Ukraine)

Special attention was given to securing the loop inside the delivery spiral during system withdrawal through the microcatheter. After testing several options, the simplest and most effective solution was selected: press-fitting the loop into the delivery spiral with a slight oversize. This method provided a stable connection without complications during retrieval and preserved the key functional advantages of our system (Figures 8A, 8B)

Tensile strength evaluation of the system components

To evaluate the safety of the developed system during microcoil repositioning and retrieval, we calculated the tensile strength of both the delivery microcable and the microcoil’s microfilaments. The microcable demonstrated a tensile strength of approximately 5 N, which was more than 4.5 times higher than the force required to rupture a platinum-iridium alloy wire that we more frequently used for manufacturing microcoils [9]. Unlike commercial systems that rely on rigid elements such as ball tips or welded joints, our design secured the microcoil solely through its own microfilaments.

In the initial configuration, we used 16 nylon microfilaments, which provided twice the tensile strength of the platinum wire. These filaments occupied only ~12% of the inner lumen of the coil, preserving its flexibility and hydrodynamic properties. In the later versions, four nylon microfilaments reinforced with 0.02 mm stainless steel wire were used to simplify the assembly process. The slight increase in axial stiffness was compensated by a controlled increase in the bending stiffness of the microcoil. A detailed analysis of this configuration lies beyond the scope of this study.

Results

Comparative Analysis of the Microcoil Delivery and Detachment Systems

**Figure 9:**
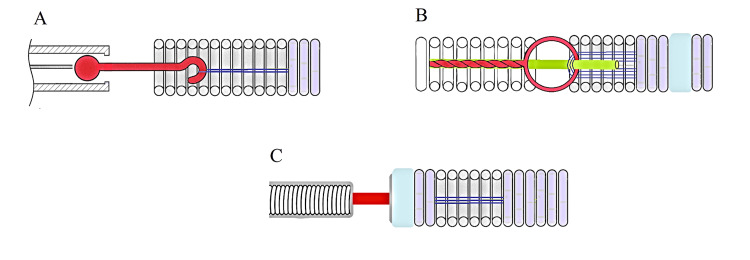
Technical sketches of three microcoil delivery systems A) Microcoil with a ball-and-socket detachment mechanism; B) Our design with a looped hinge joint; C) Microcoil with an electrolytic detachment mechanism. Image credit: Original photo sketches edited by the authors using vector editor + paint (Inkscape, Inkscape Project, open-source software).

This study analyzed three representative types of microcoil delivery and detachment systems used for embolization. The input parameters were: (1) System A: Half-tube, d=0.25 mm, Wall thickness=0.07 mm, L=10 mm; (2) System B: Two interwoven wires, d=0.04 mm, L=10 mm (dual-stranded braided microcable, 2 mm pitch); (3) System C: Solid wire, d=0.08 mm, L=10 mm (Figures 9A-9C and Tables 1, 2).

**Table 1:** Absolute mechanical stiffness parameters of the delivery systems EI: bending stiffness (N·mm²); EA: axial stiffness (N/mm).

Parameter	System A	System B	System C
Bending stiffness, EI (N·mm²)	~15.75	~0.0503	~0.4021
Axial stiffness, EA (N/mm)	~4122	~502.6	~1005.3

**Table 2:** Comparative stiffness ratios of System B vs. Systems A and C

Comparison (System B vs.)	Bending stiffness, EI	Axial stiffness, EA
System A	313× lower (0.0503 vs. 15.75)	8.2× lower (502.6 vs. 4122)
System C	8× lower (0.0503 vs. 0.4021)	2× lower (502.6 vs. 1005.3)

The obtained results aligned with the well established physical principle that the bending stiffness of a circular wire was proportional to the fourth power of its diameter [10]. It should be noted, however, that our calculations did not account for the stiffness contribution of the delivery spiral in our system, nor for certain structural components present in systems A and C, such as the polymer tubing, locking wire, or the polymer-coated coil segments, all of which may further influence its overall mechanical behavior.

Comparison of the Microcoil Detachment Systems (Locks)

The input parameters were for the systems were: (1) System A: Tube, d=0.25 mm, L=1 mm + wire, d=0.08 mm, L=1.5 mm; (2) System B: Loop, d=0.04 × 2 mm, L=1 mm + plastic rod, d=0.06 mm, L=1.5 mm; (3) System C: Wire, d=0.08 mm, L=2.5 mm (Table 3)

**Table 3:** Mechanical comparison of the microcoil detachment zones

Parameter	System A	System B	System C
Bending stiffness, EI (N·mm²)	~7.68	~0.0503	~0.4021
Total angular mobility (°)	60°	180°	0°

It must be noted that the bending stiffness (EI) characterized the resistance of the system to flexural deformation, with higher values indicating greater rigidity. The total angular mobility reflected the maximum achievable deflection without permanent deformation or failure of the detachment mechanism.

Although these calculations were physically well-grounded, their absolute values should not be overemphasized, as they are relatively small compared to the mechanical properties of the blood vessels and other components involved in the surgical procedure. However, when modeling which system characteristics were most critical in influencing a microcatheter bent at 90 degrees, the following results were obtained (Table 4).

**Table 4:** Mechanical properties of the microcoil delivery systems in a 90° microcatheter bend

Parameter	Unit	System A	System B	System C
Bending stiffness (EI)	N·mm²	0.0002327	7.25E-08	2.8E-06
Compliance (C)	mm/N	0.038	121.5	3.15
Force required for 90° bend	N	0.524	0.0036	0.0126
Outer diameter	mm	0.25	0.08	0.08
Flexibility	—	Low	High	Moderate
Tendency to deform	—	Minimal	High	Moderate

**Figure 10:**
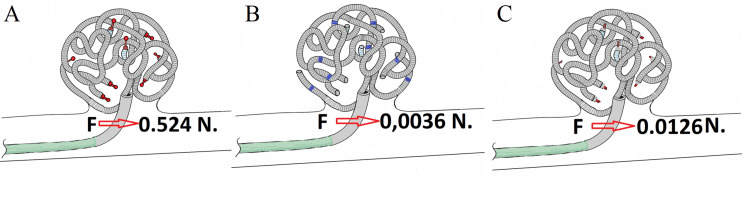
Technical sketch illustrating how the force F, depending on the delivery system type, acts on the microcatheter A) Microcoil with a ball-and-socket detachment mechanism; B) Our design with a looped hinge joint; C) Microcoil with an electrolytic detachment mechanism. Image credit: Original photo sketches edited by the authors in vector editor + paint (Inkscape, Inkscape Project, open-source software).

System B required the least force to pass through a bend and had the highest compliance, which is critically important for safe navigation in complex anatomy and tortuous vessels (Figures 10A-10C).

Supplementary Videos

The presented videos allow for a comparison of the impact on the microcatheter between a microcoil delivery system with a ball-type detachment mechanism and our flexible delivery system (Videos 1, 2).

**Video 1:** Demonstration of the microcatheter deflection under the influence of a delivery system with a ball-type detachment mechanism Original recording by the authors.

**Video 2:** Demonstration of the microcatheter deflection under the influence of our flexible delivery and detachment system Original recording by the authors.

It should be separately noted that the effect related to coil deployment was identical in both videos.

System Limitations

Temperature sensitivity: If the system, assembled at room temperature, is cooled to -5 °C, the polyethylene filament contracts by 4-5 mm, which may lead to unintentional coil detachment. This limitation can be addressed by using temperature-stable materials such as Vectran® [11]. This material can also be used as the filament of the microcoil, and considering its high tensile strength, only two filaments with a diameter of 0.04 mm would be sufficient. Although we have already developed a method for attaching such materials to the microcoil, it still requires a full set of validation tests.

Coefficient of friction: The “metal-polyethylene” pair has a coefficient of friction approximately five to six times higher than the “metal-Teflon” pair used in commercial ball-lock systems. However, despite the increased theoretical resistance during advancement, no adverse effects were observed in our tests. It is likely that the extremely low interaction forces in practice compensate for the increased friction. If needed, we have a technical solution that replaces the interface with a “polyethylene-Teflon” pair, which would offer better performance than the “metal-Teflon” [12].

## Discussion

The obtained computational data highlight the clear advantages of System B in terms of microcatheter compatibility and overall flexibility. At the same time, it may appear that the relatively simple structural modifications we introduced resulted in a noticeable improvement to a system that already functioned with high efficiency. However, in real clinical practice, enhancing one parameter, even a critically important one, is often accompanied by compromises in other essential characteristics of the system.

Nevertheless, the potential benefits of our microcoil design reasonably motivated further in vitro testing and refinement. These efforts enabled us to achieve a balanced engineering solution and present a finalized version of the delivery system. An additional advantage of the system lies in the simplicity and reliability of the detachment mechanism. Detachment occurs instantly after the removal of the locking filament, which releases the tension and allows the coil to separate by regaining its original shape. The fixation elements fold back into the delivery system and do not obstruct the detachment process. This is fundamentally different from ball-lock mechanisms, where the entire structure must be retracted into the microcatheter to release the locking element [13]. Another important benefit is that only the microcoil remains implanted in the aneurysm, with no residual locking mechanism components left behind. This minimizes the risk of mechanical irritation or potential damage to the aneurysm wall [14,15].

Although in vivo validation has not yet been conducted, we emphasize that all materials used, such as platinum, stainless steel, and polyethylene, are well-characterized and widely implemented in commercial neuroendovascular devices. Our in vitro tests confirmed that the mechanical characteristics of the system, including detachment reliability and tensile strength, fully complied with current standards. The implanted element is a standard platinum microcoil with a modified proximal microfilament loop, so no additional biocompatibility issues are anticipated. Any future in vivo use of the system will, of course, follow applicable legal and ethical regulations.

Previous studies have shown that complications such as premature coil detachment, microcatheter kickback, or even aneurysm rupture may result from excessive stiffness and friction of endovascular tools, as well as reduced control during coil implantation [16]. In particular, under conditions of complex vascular anatomy, surgeons may intentionally detach the microcoil inside the microcatheter to avoid losing precise tool positioning [17]. This further emphasizes the need for continued engineering improvements, specifically in microcoil delivery.

It should also be noted that during embolization, the forces acting on the microcatheter and aneurysm wall depend not only on vascular anatomy, the properties of the delivery system, and the microcoil itself, but also on the degree of aneurysm filling with coils [18,19]. As packing progresses, loads on the microcatheter increase, its position within the aneurysm dome shifts, and interactions with the previously implanted coils change. If the delivery system lacks sufficient mechanical performance, these factors may complicate the final stages of coiling and increase the risk of procedural complications.

These clinically significant challenges were among the main factors that prompted the development of our device. Although it has already reached the final prototype stage, we continue to work on simplifying its design, improving functional performance, and reducing manufacturing costs. In particular, friction during advancement through the microcatheter was reduced by more than half, improving control during coiling. The number of manufacturing steps was reduced from seven to three, significantly increasing production efficiency. At the same time, a new microcoil configuration is being explored, offering better adaptation to the aneurysm wall and improved shape retention due to the use of nickel-titanium alloys (nitinol). This configuration increases the external contact area by 50% without changing the overall dimensions of the coil. These directions may lead to breakthrough solutions both in the coil design and in the delivery system, potentially simplifying the surgical procedure and enhancing the safety and effectiveness of endovascular aneurysm treatment.

## Conclusions

This study presents the design, comparative analysis, and in vitro evaluation of a new mechanical delivery and detachment system for microcoils intended for endovascular embolization of cerebral aneurysms. The proposed system demonstrated high flexibility, improved compatibility, and minimal mechanical impact on the microcatheter, both in the intracranial segment and in the detachment zone. These features may help simplify the procedure and reduce the risk of surgical complications, particularly in cases of complex vascular anatomy or limited endovascular access due to other clinical factors. The system may also improve the finalization of the coiling procedure and ensure the required level of microcoil packing within the aneurysm.

Unlike traditional ball-lock detachment mechanisms, which require partial retraction of the system into the microcatheter for release, the proposed looped hinge configuration provides not only flexibility and mobility but also immediate and clean detachment of the microcoil without leaving any residual components inside the aneurysm cavity. Bench testing confirmed the reliability, reproducibility, and safety of this mechanism. The identified technical limitations, specifically, the shrinkage of the polyethylene filament at low temperatures and the increased friction compared to Teflon-based systems, can be effectively mitigated through targeted material selection. The use of biocompatible components (platinum, polyethylene, and stainless steel) further supports the potential of this system for future clinical application following in vivo validation.

Although the current prototype has already been finalized and tested in vitro, the system may serve as a foundation for further improvement, simplification, and cost reduction of tools used in microcoil embolization. Its design addresses key mechanical challenges and holds potential to enhance the safety and controllability of endovascular procedures in the treatment of aneurysms.

## Figures and Tables

**Figure 11:**
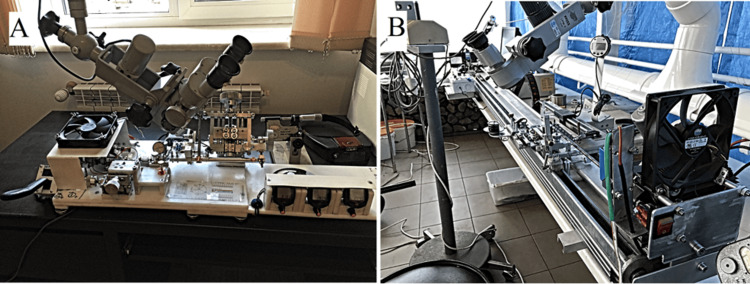
Device for microcoil fabrication by (A) forming and (B) winding Image credit: Original work by the authors.

**Figure 12:**
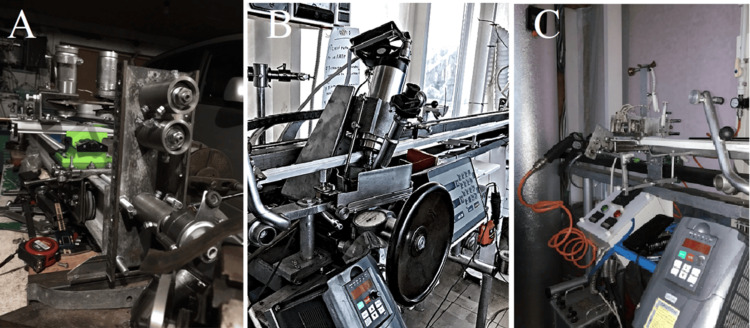
A) Two-spindle cylindrical grinding machine, B) single-spindle cylindrical grinding machine, and C) device for wire straightening Image credit: Original work by the authors.

**Figure 13:**
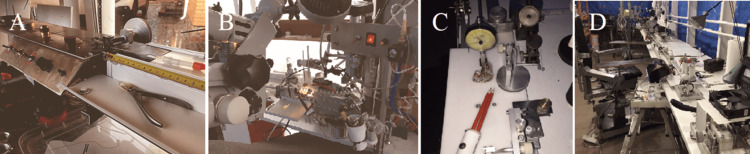
Device for (A) inserting the filament into the delivery system; (B) micro-welding of metals; and (C) fixing microfilaments to the microcoil; along with (D) workstation and device for assembling the delivery system and fixing the microcoil Image credit: Original work by the authors.

**Figure 14:**
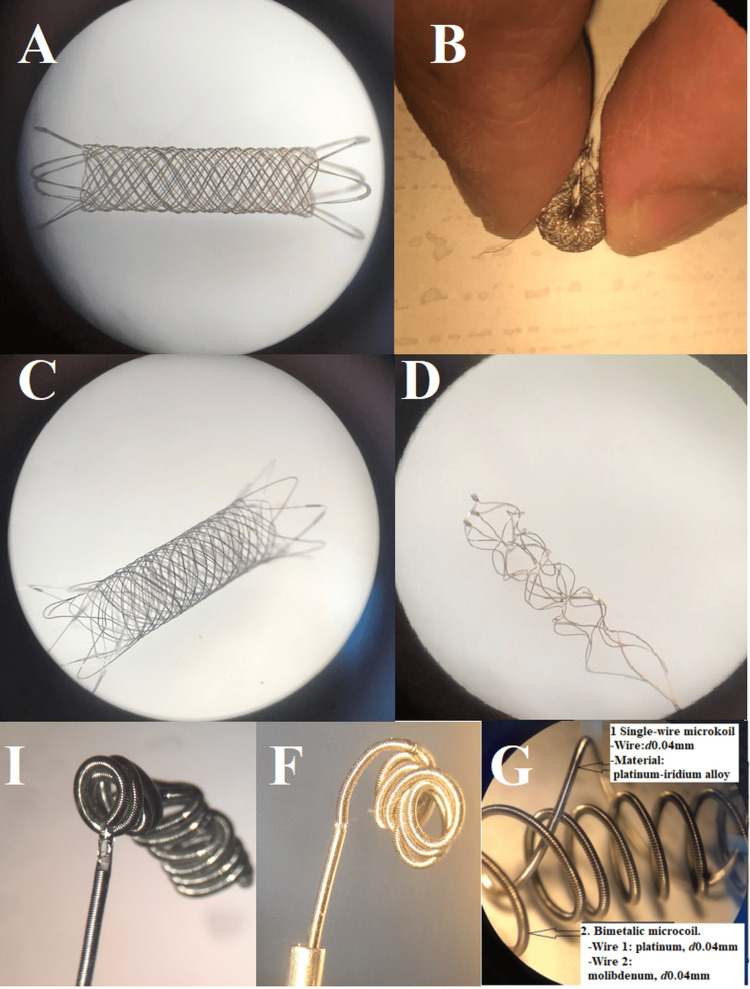
Microscopic photos of our experimental devices A. Extracranial stent for deformed and tortuous vessels with anti-embolic braiding; B. Demonstration of the stent flexibility; C. Intracranial stent; D. Thrombectomy device;  I. Microcoil mounted on a flexible delivery system with instant electrolytic detachment; F. Microcoil presented in this study; G. Bimetallic microcoil. Image credit: Microscopic photo taken by the authors with the Carl Zeiss microscope (Ukraine).

## Appendices

Supplementary note: Ongoing experimental developments

The device designs listed below are currently at the stage of experimental development and are not part of the main scope of this study. Their purpose is to improve endovascular tools in order to simplify surgical procedures and increase their predictability and effectiveness. Some of the presented configurations may have greater clinical potential than the system described in the main part of this article. We understand the conservatism of the medical device market; however, these concepts may serve as a foundation for future research and technical collaboration.

Prospective development of a bimetallic microcoil for cerebral aneurysm embolization (NiTi + Ta)

This microcoil design demonstrates 20-30% greater stiffness compared to platinum-based equivalents. Due to the specifics of the manufacturing process, it has an approximately ~1.5× larger external contact area while maintaining standard geometric dimensions. Like microcoils made from nitinol-core wire, it possesses shape memory properties; however, in our design, this effect is approximately twice as strong.

A unique feature of the microcoil is the ability to selectively increase the stiffness of individual turns in the final 3D configuration by eight to 16 times, which may be useful for the development of new framing microcoils. The estimated material cost for manufacturing this microcoil is approximately half that of platinum-based alternatives.

Two Types of Thrombectomy Devices

Stent retriever, compatible with microcatheters with an internal diameter of ~0.4 mm, made of 16 nitinol wires uniformly braided across the entire cross-section. For radiopacity, each wire is helically wrapped along its entire length with a 0.04 mm tungsten wire at a 0.08 mm pitch, forming a composite element. This configuration ensures effective thrombus capture and retention due to asymmetrically open mesh cells in the braid. Preliminary estimates indicate that the thrombus contact area is approximately five times greater than that of most commercial retrievers. The design minimizes the risk of vascular wall injury and reduces the number of required passes.

Bimetallic “hedgehog spiral”, made of nitinol and tungsten, has a double-layer spiral structure with an asymmetric phase shift that forms outward-pointing elliptical loops. This geometry provides a 3-4× larger contact area with the thrombus compared to standard devices. Thrombus retention is achieved through a combination of the shape memory effect and a special tightening filament that constricts and holds the loops in a capture-effective configuration.

Additional Systems

Monorail microguidewire tip deflection system: An accessory designed to improve steerability of the distal tip of the microguidewire in complex vascular anatomy.

Stent delivery system with proximal deployment control: Designed for precise positioning and controlled deployment of extracranial braided nitinol stents in anatomically challenging areas (e.g., bends, loops).

Instant electrolytic microcoil detachment system: A flexible microcoil delivery system with electrolytic detachment (GDS-type), developed for instant and predictable coil release under fluoroscopic guidance (see Figures 14A-14G).
